# The elusive power of the individual victim: Failure to find a difference in the effectiveness of charitable appeals focused on one compared to many victims

**DOI:** 10.1371/journal.pone.0199535

**Published:** 2018-07-18

**Authors:** P. Sol Hart, Dan Lane, Sedona Chinn

**Affiliations:** Department of Communication Studies, University of Michigan, Ann Arbor, MI, United States of America; Queen Mary University of London, UNITED KINGDOM

## Abstract

Previous research has offered conflicting findings regarding the influence of help appeals that feature an individual victim compared to a group of victims. Studies examining *emotional responses* and *donation behavior* have generally found that help appeals focusing on a single victim elicit more prosocial responses, while studies examining *policy support* have found the opposite. The present studies investigate these effects while addressing potential confounds that may have arisen in previous research. Study 1 (*N* = 924) compares the effects of help appeals that focus on either a) an identified individual, b) an identified group, c) statistics describing many individuals, or d) statistics paired with an individual. Study 1 also examines how the location of the individual(s) in need moderates observed effects. Study 2 (*N* = 1,085) compares the effects of help appeals that describe either an identified individual or statistics about many individuals, while fully crossing the text manipulation with either a) no imagery, b) an image of an individual, or c) a map indicating areas of poverty. In both Study 1 and Study 2 no significant differences were found between the individual and the group conditions.

## Introduction

Research on the psychological processes underlying prosocial decision-making informs the strategies that journalists, activists and philanthropic organizations use to garner sympathy and support for those in need. Such is the case with a collection of studies demonstrating that as the number of individuals in need increases, emotions and helping behavior may decrease [1–3]. This research suggests charitable appeals that draw attention to the plight of a single individual, rather than masses of affected people, will be more effective in promoting helping behavior. While charitable organizations have traditionally emphasized how social problems affect large swaths of society, research supporting the efficacy of messages with a single, identifiable victim has helped usher in a new era of philanthropic storytelling centered around the power of individual stories [4,5].

Despite the enthusiastic reception of these findings among journalists and nonprofit communicators [6], there have been some inconsistencies in this research which have not been fully reconciled. For example, studies examining how depictions of victims may alter policy preferences, rather than donations, have found that focusing on individuals can lead to *less* support for policies to help those in need compared to stories that present statistics about the overall issue [7–9].

Given these conflicting findings and the public interest in the power of individual stories, this paper attempts to clarify the effectiveness of portraying an individual victim, as opposed to many victims, in charitable appeals. In two experimental studies, we examine how appeals featuring different numbers of victims influence emotional responses, policy support, willingness to make donations, and willingness to volunteer. In doing so, we address potential confounds that exist in previous work and test factors upon which this effect has been shown to be contingent, including group membership of the victim (Study 1) and imagery (Study 2).

## Literature review

### The psychological power of the individual victim

A rational, normative ideal might posit that all lives hold equal value [2]. Under this paradigm, as more lives are at stake, the willingness to expend resources to help those in need would increase in a corresponding manner. Yet studies examining how individuals respond to help appeals have found that not only do individuals fail to follow this normative ideal, but that they often follow an opposite pattern of helping. Previous research has termed this phenomenon “psychic numbing” [2] or “compassion fade” [3,10], which refers to a predisposition to have stronger emotional responses and a greater willingness to help a single individual in need, compared to a group of individuals or many in need.

Slovic [2] proposed a model of helping behavior to explain the psychic numbing effect. This model holds that when individuals are presented with information about an individual in need they are better able to pay attention to [11,12] and elicit mental imagery about the condition of the individual [13], compared to when they are presented with information about multiple individuals or statistics of many in need. The result of this increased attention and mental imagery is an increase in feelings, or affect, which drives subsequent helping behavior [2].

Affect is the general feeling that something is good or bad [14,15], and plays a critical role in assigning meaning to information and in driving behavior [16]. When individuals have an affective association with an object, the object can more easily be incorporated into decision-making [17]; in other words, affect helps individuals understand the world around them and informs intuitive judgements on how to act.

The role that affect plays in decision-making can be explained, in part, by dual process theories of thinking [18]. Affect is most closely related to what is known as system 1, or the experiential system, as compared to system 2, or the analytic system [19]. Through system 1, affect drives the immediate, intuitive assessment of a situation [14]. While an individual may subsequently use a more deliberative approach to assess that situation, such deliberations are typically colored by the initial assessment made through the affective evaluation [20]. In many cases affect can play a more prominent role in judgment and decision-making than objective reasoning [14].

Under Slovic’s model [2], stronger affective responses are likely to motivate prosocial behavior. This relationship has been observed in studies demonstrating an “identified victim effect [1,21,22].” Kogut and Ritov [1,21] found that feelings of emotional distress and donations increased in response to a help appeal for children with cancer when help was requested for a single, identified child in need, rather than a group of 8 identified children in need. Identifying information about the individual(s) in need (name, age, and photograph) was critical for this effect to occur. Without this identifying information there was no difference in helping behavior between the individual and the group. Subsequent research found this effect held when an appeal with one child in need was compared to an appeal with two children in need. Even when only two children were shown, both hypothetical and real donations were less than when one child was shown, though this effect was mitigated if the children were presented as related to one another [3].

A closely related line of research [22] has investigated how people respond to an individual in need compared to statistics about millions in need. This differs somewhat from the previously discussed research, as statistics were utilized to represent the larger group rather than a finite number of identified individuals. The general pattern of the previously discussed research was replicated; individuals had stronger emotional responses and were willing to donate more for a single individual in need compared to millions in need. When individuals are faced with vast numbers of people in need, they may become emotionally overwhelmed by the thought they cannot help all those in need [23]. The resulting negative affect may be a demotivating force, resulting in less helping behavior [23]. Taken together, this literature suggests that appeals featuring a single individual in need may be more effective than those featuring multiple individuals, groups, or statistics.

### When an individual victim discourages helping behavior

While previous work has generally found that a help appeal with a single identified individual can lead to greater donations, literature examining policy responses has generated a different pattern of results. For example, Iyengar [8,9] examined how individuals respond to discussions of poverty in the United States, looking specifically at how support for welfare can vary from exposure to a narrative about an impoverished individual (termed an episodic frame) compared to statistics about poverty in the U.S. as a whole (thematic frame). Contrary to research on the identified victim effect and compassion fade, Iyengar found that participants were more supportive of welfare policies when presented with many in need, rather than an individual.

In the environmental domain, Hart [7] investigated how individuals responded to a news story about one polar bear in need or many polar bears in need. In terms of outcomes, Hart did not focus on donations, but rather changes individuals might make in their own lives to address global warming (e.g. installing energy efficient lighting in the home). Hart found no difference between the conditions in willingness to take individual action, but did find that participants had more support for climate mitigation policies after reading about the risks climate change poses to many polar bears, compared to a single polar bear.

Additional studies examining donations have found similar results [24,25]; portraying an individual victim had the potential to increase attribution of responsibility to the individual and thus reduce the likelihood that the message receiver would take action to help the individual.

### Present research

The goal of the present research is two-fold; 1) to reconcile the conflicting findings in the reviewed literature, and 2) to eliminate potential confounds that have appeared in previous research in this area. First, as we have noted, there is empirical evidence that portraying either a single victim *or* many victims can alternately result in greater helping behavior. We suggest that conflicting findings in previous work may stem from differences in the dependent variables examined. Previous studies examining responses to help appeals for individual and many victims typically only focus either on emotional responses, donation behavior, or policy support, but rarely examine all of these measures together. This makes it difficult to determine whether differences in findings are simply a result of the type of dependent variable studied. Accordingly, in Study 1 we test for effects on all of these outcome measures.

There are also important differences in the ways in which studies have manipulated experimental stimuli to portray many victims. For example, the “many” condition in prior studies has varied with respect to the number of individuals portrayed and the use of statistics in the help appeal. We are not aware of previous research that has directly examined how individuals may respond to appeals with an individual, a group of identified individuals, or statistics about many in comparison with each other. Thus, in order to help disambiguate the influence that the number of individuals in need may have on helping behavior, we include multiple representations of “many” individuals as experimental conditions in Study 1.

Finally, while the work of Small, Loewenstein, and Slovic [22] offer some of the most widely cited evidence of the efficacy of messages featuring identified individuals, it is important to note several potential confounds that may have impacted their results. In the Small, Loewenstein, and Slovic study, the individual victim condition 1) included an image, 2) was in a narrative format, 3) discussed how the money would directly help the individual in need, and 4) focused solely on a child. In contrast, the many victims condition 1) included no image, 2) contained bullet pointed statements rather than a narrative, 3) did not include information about how donations would help those in need, and 4) referred to larger populations that included adults rather than focusing solely on children. It is possible that these differences could have driven some of the observed effects. To address this possibility, we conduct an adaptation of Small, Loewenstein, and Slovic’s experiment in Study 2 while addressing potential confounds.

## Study 1

As we have noted, there have been conflicting findings regarding the effect of portraying one vs. many victims, with studies using donations as the dependent variable generally finding portrayals of one individual to be more effective [1,2,13], while studies focusing on policy support generally finding portrayals of a group to be more effective [7–9]. To the best of our knowledge, these variables have not previously been examined in the same study. Study 1 attempts to clarify the effects of appeals featuring individual victims and groups of victims on these respective dependent variables.

Further, studies in this area typically compare an identified individual to either a group of identified individuals [3], to an identified individual *and* a group [26], or to statistics [22]. In addition, work has compared how the inclusion of statistical information influences the impact of help appeals featuring groups and individuals [27]. Previous studies have not included these various victim presentations in the same between-subjects design. Study 1 incorporates these factors to better understand how emotional responses and prosocial behavior may shift across different numbers of people (an identified individual, a group of identified individuals, statistics about millions of individuals, and statistics paired with an identified individual).

Finally, Study 1 performs an exploratory investigation into the role that the location of the victim(s) may play in driving different responses. Previous research [28,29] found that the identified victim effect is more likely to occur when the victim is someone that participants have an affinity for. Self-categorization and identification with the victim affected responses to appeals for help [30,31]. Thus, emotional responses and helping behavior may vary depending on the location of the victim, with empathy [32,33] and affect [34,35] increasing the allocation of resources toward targeted victims. Iyengar’s [8,9] work finding greater policy support in response to thematic representation of those in need compared to identified individuals may have been affected by the fact that the victims shared a national identity with those surveyed, which has not always been the case in studies examining donation behavior [2,22].

Overall, we predict that consistent with previous research on the identified victim effect emotional responses and donations will be greater for the identified individual. We also predict, based on research by Iyengar [8,9] and Hart [7], that support for policy will exhibit the opposite pattern of effects. For both of these manipulations, we explore the research question of how the location of the victims may moderate the predicted effects. Overall, Study 1 examines how these respective manipulations may interact, and how the number and location of individuals in need may impact emotional responses, willingness to donate, policy support, and willingness to volunteer.

### Method

#### Participants

One thousand one hundred and fifty-four Americans were recruited using Amazon’s Mechanical Turk for a survey on how individuals respond to media messages. All participants consented to participate in a procedure approved by the Institutional Review Board at the University of Michigan (HUM00102853). Two hundred and thirty participants failed one of a series of attention check measures and were removed from the sample, resulting in a final sample of 924 (*M*_age_ = 35.39, *SD*_age_ = 11.92; 47.20% Male). The majority of participants self-identified as White or Caucasian (79%), followed by Black or African American (6.8%), Hispanic (5.60%), Asian or Asian American (4.7%), and other racial groups (3.9%). Participants on average identified as slightly liberal (*M* = 3.27, *SD* = 1.59) on a seven-point scale ranging from (1) very liberal to (7) very conservative. Participants had a median education level of “some college or no degree” and a median income of $40,000 - $59,999.

#### Simulated donation paradigm

After providing informed consent and demographic information, a simulated donation paradigm was administered in which participants were told they had been entered into a “Participant Appreciation Program” drawing and were eligible to win $50 in cash to be directly deposited into their Amazon account if they were the winner. This provided a potential sum of money with which participants could indicate they would use to make a donation if they won the drawing.

#### Experimental stimuli

Next, participants were randomly assigned to view a charitable appeal from a fictional non-profit “Helping Hands,” which provides both life-saving assistance and long-term support to women living in poverty. Appeals were manipulated to vary the number of individuals in need (one, five, many, or one and many) and geographic location (United States or Kenya). The number of individuals was indicated through both text (e.g., “Ruth is a single mother” vs. “millions of single mothers”) as well as with pictures (a photo of a single woman, a photo of a group of five women, a map of the target country or a map of the target country alongside a picture of a single woman). Although early work suggested that photos could have a negative effect on donation behavior [36], more recent research has emphasized that inclusion of a picture of an individual in need provides identifying information that is critical to identified victim effects [1,21,34]. Location was manipulated by changing the country name in the text and the map of the country in conditions that included a map. All appeals described how the target individual(s) struggled to provide their children with decent food and medical care and faced job insecurity and difficulty finding work. The appeals also indicated that without help the individual(s) and their children would be at an increased risk of food insecurity, homelessness and desperation. Appeals featuring one or five individuals provided first names for each woman. To maximize ecological validity, all experimental stimuli were created to look like realistic charity appeals–a professional graphic designer constructed the stimuli in the format of existing charitable appeals (see S1–S8 Figs for full stimuli).

### Variables

#### Emotion

Previous research has taken different approaches to measuring emotional responses to individual victims in need, often combining measures of negative affect, moral evaluation and mood management into a single index [22,37]. Consequently, we included two set of emotion measures in order to account for possible variation due to measurement strategy. The first set of measures included items from Small, Loewenstein, and Slovic [22] and asked participants to evaluate their feelings in regards to the charity appeal. Participants were asked to indicate on a 5-point scale that ranged from (1) Not at all to (5) Extremely; 1) “How upsetting is this situation to you?” 2) “How sympathetic did you feel while reading the description of the cause?” 3) “How much do you feel it is your moral responsibility to help out with this cause?” 4) “How touched were you by the situation described?” and 5) “To what extent do you feel that it is appropriate to give money to aid this cause?” All items were combined into an index of *feelings* (*M =* 3.29, *SD =* .98, α = .90). While we utilized the original scale from Small, Loewenstein, and Slovic, we also note that some of the items appear to measure different theoretical constructs (e.g., how upsetting the situation is vs. perceived moral responsibility to act); therefore, we included a set of measures that exclusively assessed emotional response. Six bipolar items, each measured on a six-point scale, asked respondents how they felt while reviewing the charity appeal (Not at all sad/Sad, Not at all happy/Happy, Not at all upset/Upset, Not at all distressed/Distressed, Not at all sympathetic/Sympathetic, Not at all guilty/Guilty). All items (with Happy reverse coded) were combined into an index of *negative affect* (*M =* 3.96, *SD =* 1, α = .82). The feelings and negative affect scales were highly positively correlated (*r* = .72, *p* < .001).

#### Willingness to donate

To assess participants’ willingness to donate to the charitable organization featured in the appeal, we first asked if they would be willing to donate some of their winnings from Participant Appreciation Program if they were to win to the charity. This response was coded as a binary variable (“No” = 0; “Yes” = 1) and labeled *donation willingness* (68.3% “Yes”). We next asked participants to indicate how much they would be willing to donate from $0 to $50 and labeled this variable *donation amount* (*M =* 12.83, *SD =* 13.94).

#### Policy support

Participants were asked to indicate on a six-point scale ranging from (1) Strongly oppose to (6) Strongly support, their support for three potential congressional bills relating to single-mothers in the country mentioned in the charitable appeal they reviewed. The first two bills called for an increase in federal funding for 1) immediate emergency assistance for food and shelter and 2) for job training seminars and counseling, respectively. The third bill called for balancing the federal budget through cutting a program that provides supplemental nutrition assistances for single mothers. This third bill was reverse coded and then all three measures were combined into an index of *policy support* (*M =* 4.17, *SD =* 1.17, α = .67).

#### Volunteering

We assessed participants’ willingness to volunteer for the charitable organization. On a five-point scale ranging from (1) Not at all willing to (5) Very willing, participants indicated how willing they were to engage in 5 volunteer activities, including 1) “donate used clothes to a family living in poverty,” 2) “advocate for anti-poverty efforts at my state capitol,” 3) “write a letter to my congressperson in support of anti-poverty programs,” 4) “attend a peaceful march in support of the assistance of low-income single mothers,” and 5) “donate school supplies to a specific child living in poverty.” All measures were combined into an index of *willingness to volunteer* (*M =* 3.82, *SD =* 1.15, α = .86).

### Results

In the supplementary files, Table A in S2 File provides descriptive statistics by condition for the dependent variables in Study 1 and Table B in S2 File provides the overall zero-order correlations between dependent variables.

Study 1 was analyzed using a factorial ANOVA to investigate the impact of location and the number of victims in need on the dependent variables of feeling, negative affect, donation, policy support, and willingness to volunteer. The results reported below do not include respondents who were excluded for completing the experiment too quickly (less than 4 minutes) or for taking too long (more than 30 minutes); the pattern of these results does not differ from an analysis that includes all respondents regardless of time for completion.

Looking first to the impact of altering the number of individuals at risk, we found no significant main effects of the treatment conditions on the dependent variables of feeling, *F*(3, 916) = 1.45, *p* = .23, negative affect, *F*(3, 916) = .52, *p* = .67, donation amount, *F*(3, 916) = .95, *p* = .41, policy support, *F*(3, 915) = .65, *p* = .58, or willingness to volunteer *F*(3, 916) = .29, *p* = .83.

Looking next to the impact of where the victim was located, no significant effects were found for the dependent variables of negative affect *F*(1, 916) = 1.81, *p* = .18, and donation amount *F*(1, 916) = .76, *p* = .38. Significant differences were found for the dependent variables of feeling *F*(1, 916) = 10.71, *p* = .001, policy support *F*(1, 915) = 170.82, *p* < .001, and willingness to volunteer *F*(1, 916) = 17.32, *p* < .001. Levels of feeling, policy support, and willingness to volunteer were higher when individuals in need were located in the United States.

Finally, looking to interactions between the number of individuals at risk and the location of the individuals, there were no significant interaction effects for the dependent variables of feeling, *F*(3, 916) = .994, *p* = .40, negative affect, *F*(3, 916) = 1.37, *p* = .25, donation amount, *F*(3, 916) = .962, *p* = .41, policy support, *F*(3, 915) = .966, *p* = .41, or willingness to volunteer *F*(3, 916) = .49, *p* = .69.

### Discussion

Overall, the results from Study 1 fail to support the hypothesis that manipulating the number of individuals in need in a charitable appeal will alter emotional responses and helping behavior. The results do reveal mixed evidence that the location of the individual in need can alter emotions and helping behavior–no effect was shown for negative affect and donation amount, but main effects were revealed for feeling, policy support, and willingness to volunteer, with individuals more willing to help U.S. based victims than Kenya based victims. While previous research suggested that the identified victim effect might only emerge when victims are socially close to the respondent [38], no interactive effects were found based on the location of the victim. Overall, the failure to find significant differences between the conditions for the number of victims raises questions about the robustness of effects driven by changing the number of victims in help appeals.

Several differences between the stimuli used for this study and previous work are worth noting. First, in order to have a condition that would be realistic for the U.S. location, we discussed individuals living in severe poverty, but not in immediate risk of dying due to starvation [2]. It is possible that showing individuals in need who were in very bad, but not extremely dire, situations could have influenced the results.

A second difference is that in this study the victims were mothers and their children. This approach may have attenuated the impact in the individual condition, as there was not a single individual by themselves in need. In addition, the results may have been impacted by the imagery utilized, as different images (an individual, a group of individuals, a map, and a combination of an individual and a map) were used for the corresponding text conditions. While the use of these images was intended to amplify the differences between the conditions, it may have unintentionally drawn attention away from differences in the text.

In light of the limitations of Study 1, we conducted a second study to focus more closely on how changing the number of individuals in need may impact responses for our variables of interest. Our goal in Study 2 was to utilize a stimulus that more closely approximated that used by previous research [2,22] while exploring how different appeals might influence emotional responses, individual helping behavior, and policy support.

## Study 2

Study 1 failed to find a significant impact of varying the number of victims in a help appeal. Study 2 was designed to address two potential issues with Study 1: 1) the combination of mothers and their families in the text, rather than individuals alone and 2) the varying of the image associated with the different text conditions. In order to minimize differences between our study and previous work, we used language for the individual condition that was very similar to language used previously in Small, Loewenstein, and Slovic [22], which calls for aid for children in need in Africa.

To avoid the potential image confound in Study 1, we fully crossed imagery and text in Study 2. As an identified group of individuals was not as amenable to this fully crossed design, we focused on comparing appeals portraying a single individual in need to appeals portraying a large number of people in need. Finally, potential confounds in the original Small, Loewenstein, and Slovic [22] study are minimized in Study 2 by matching the text across conditions in terms of imagery, narrative format, information about how the donations will be used, and number of victims.

### Method

#### Participants

All participants were recruited through Qualtrics panels for a brief survey on how individuals respond to media messages. Qualtrics recruits participants through e-mail sign-up, web banners, social media, and invitation only methods. Quota targets were set to ensure balance within gender (50% male / 50% female), age (33% 18–34 years, 33% 35–54 years, 33% 55+ years) and education categories (42% HS diploma or less, 29% some college or no degree, 29% bachelor’s degree or higher). One thousand three hundred and twenty participants were recruited and provided informed consent to the same study procedures as were used in Study 1 (HUM00102853). Two hundred and thirty-five participants failed a time check measure and were removed from the sample, resulting in a final sample of 1,085 (*M*_age_ = 44.92, *SD*_age_ = 16.05; 48.60% Male). The majority of participants self-identified as White or Caucasian (80.6%), followed by Black or African American (6.9%), Hispanic (5.8%), Asian / Asian American (3%), and other (3.7%). Participants on average identified as slightly liberal (*M* = 3.96, *SD* = 1.66) using the same scale as in Study 1. Participants had a median education level of “some college or no degree” and a median income of $40,000 - $59,999.

#### Procedure and stimuli

The same simulated donation paradigm and similar outcome measures from Study 1 were used, including *feelings* (*M =* 3.19, *SD =* 1.03, α = .92), *affect* (*M =* 4.07, *SD =* 1.11, α = .85), *donation willingness*, (“Yes” = 42.8%), and *donation amount* (*M =* 7.89, *SD =* 13.71) *Willingness to volunteer* used similar, but not identical, measures from study 1; on a six-point scale ranging from (1) Very unwilling to (6) Very willing, participants indicated how willing they were to engage in 4 volunteer activities, including 1) “donate school supplies”, 2) “staff an information table at a local [organization’s name] event”, 3) “Sign an online petition in support of international anti-poverty efforts”, and 4) attend a peaceful march in support of international poverty awareness. These items were combined into a single willingness to volunteer scale (*M =* 3.40, *SD =* 1.42, α = .90). For policy support, participants were asked to indicate on a six-point scale ranging from (1) Strongly oppose to (6) Strongly support, their support for increasing government funding: 1) for immediate emergency assistance for food and shelter, and 2) to purchase uniforms and receive a healthy balanced meal at lunch time for the victim/victims. These two items were combined into an index of *policy support* (*M =* 3.87, *SD =* 1.55, *r* = .92)

In order to closely replicate the experimental conditions under which previous effects have been found, we adapted stimuli from Small, Loewenstein, and Slovic [22], in which a real charitable organization called “Doctors Without Borders” solicited donations to help the plight of a starving child/children in Africa. We used the same story (e.g. child/children facing starvation in Africa), but standardized the wording and added image as a fully crossed factor (See Figures A-E in S1 File for full stimuli). Ultimately, participants were randomly assigned to view a charitable appeal that varied in number of victims mentioned in the text (one vs. many) and imagery (image of an individual child, image of a country map or no image). The wording for the one and many conditions was identical except for the individual child’s name, “Rokia,” was replaced with “girls across the East African country of Kenya” in the many conditions. We note that previous work [22] used Mali as the country of residence for the individual in need, but since 2012 insurgent groups have been fighting against the Malian government for independence in Northern Mali. We were concerned that this conflict might affect the results, and instead chose Kenya as the country of residence, which has high levels of poverty, but was politically stable at the time the study was conducted. Unlike Study 1, location was not manipulated.

To ensure that responses to conditions that included a photo of an individual child were not biased by attributes of any specific child, we stimulus sampled photos of three different children. These photos were selected from a larger set of photos, which had been pilot tested and were not statistically different from each other on ratings of perceived attractiveness, warmth, sadness, and the negative affect elicited.

### Results

In the supplementary files, Table C in S2 File provides descriptive statistics by condition for the dependent variables in Study 2 and Table D in S2 File provides the overall zero-order correlations between dependent variables.

As with Study 1, Study 2 was analyzed using a factorial ANOVA to investigate the impact of imagery and the number of victims in need for the dependent variables of feeling, negative affect, donation, policy support, and willingness to volunteer. As with Study 1, the results reported below do not include respondents who were excluded for completing the experiment too quickly (less than 4 minutes) or for taking too long (more than 30 minutes); the pattern of these results does not differ from an analysis that includes all respondents regardless of time for completion.

Looking first to the main effects of imagery, no significant effects were shown for the dependent variables of feeling, *F*(2, 1079) = .53, *p* = .59, negative affect, *F*(2, 1079) = .68, *p* = .51, donation amount, *F*(2, 1079) = .860, *p* = .42, policy support, *F*(2, 1079) = .89, *p* = .41, or willingness to volunteer *F*(2, 1079) = .114, *p* = .89.

Looking next to the main effects of the number of victims in need, no significant effects were revealed for the dependent variables of feeling, *F*(1, 1079) = .053, *p* = .82, negative affect, *F*(1, 1079) = 1.41, *p* = .24, donation amount, *F*(1, 1079) = .002, *p* = .96, policy support, *F*(1, 1079) = 1.48, *p* = .22, or willingness to volunteer *F*(1, 1079) = .76, *p* = .38.

Finally, looking to interactions between the number of victims at risk and imagery, no significant effects were found for the dependent variables of feeling, *F*(2, 1079) = 2.30, *p* = .1, negative affect, *F*(2, 1079) = 1.79, *p* = .17, donation amount, *F*(2, 1079) = .90, *p* = .41, policy support, *F*(2, 1079) = 1.16, *p* = .31, or willingness to volunteer *F*(2, 1079) = 2.34, *p* = .1.

As the stimuli for Study 2 most closely approximated some of the seminal research examining this effect [22], we also conducted a one-way ANOVA for all of the conditions in comparison with each other (not grouped by text or image) to see if the grouping by text and image may have obscured differences between some of the conditions. Using this approach, neither the ANOVA nor Sidak post-hoc comparisons were significant for any of the dependent variables.

### Discussion

As with Study 1, Study 2 did not reveal evidence to support the prediction that altering the number of victims in need will shift emotional responses or predispositions for helping behavior. Study 2 also did not find evidence that the presence or absence of imagery impacts the dependent variables under consideration.

It is important to note that the present study is not a direct replication of the previous research. As mentioned above, the stimulus for Study 2 was created to mirror much of the language of initial findings in this area [22], while using a narrative for the many condition and fully crossed imagery to avoid some of the confounds of the previous research. In doing so, we attempted to extend the previous research into additional dependent variable domains (i.e., to examine feeling, willingness to donate, policy support, and willingness to volunteer in the same study). We note that while the feeling questions were the same as those used in previous research, the donation questions were offered in response to the potential to win a lottery, rather than a direct donation with money already received, and the policy context is different from previous work that focused on policy support in a U.S. context [8, 9]. It is possible that differences in the stimuli or dependent variables between this study and previous work [22] is the reason that significant effects are not observed in Study 2. Nonetheless, as with Study 1, Study 2 raises questions about the robustness of previous research that found differences in the impact of using an individual compared to many victims in charitable appeals.

## General discussion

In both Study 1 and Study 2 we failed to find significant effects of altering the number of victims in charitable appeals. Altering the number of people in need did not lead to significant differences in feelings, negative affect, willingness to donate, willingness to volunteer, or policy support. Further, no effects emerged when the role of imagery was examined as an additional factor. The results presented here are not consistent with either the compassion fade effect or previous work looking at policy support finding an opposite effect. This suggests effects driven by focusing on an individual or many individuals may be contingent upon factors yet to be clearly elucidated and that the use of findings in this area to produce strategic communication should be undertaken with caution.

We note that our study is not the first to raise questions about the robustness of the effect of focusing on a single individual. For example, Lesner and Rasmussen [39] found no differences in responses to direct mail solicitations portraying an identifiable victim or statistics about victims. Maier [40] found little effect of focusing on an individual (vs. many) in need on the likelihood that news readers would comment, like, and share stories. Rather, topic and geographical proximity of the story played a large role in drawing interest and sharing behavior. Maier’s results echo our finding in Study 1 that, while there was no effect of number of victims, participants demonstrated favorability towards helping individuals in the U.S., compared to Kenya, with higher levels of feeling, policy support, and willingness to volunteer in response to appeals featuring Americans in need. Additionally, previous research indicates that whether the identified beneficiary is personally known to the participant [41] or shares a social identity with the participant can influence willingness to offer assistance [28–31]. These findings are a reminder that there are other strong determinants of helping behavior apart from the psychological influence of the number of victims.

While we argue that the results from the present studies highlight substantive issues in this area of research worthy of further examination, it is important to consider several alternative explanations for why altering the number of victims in need and the presence or absence of imagery did not drive significant effects. Looking first to the variables used for measurement, the outcome variables examined here captured self-reported behavior and predispositions, rather than observed behavior; it is possible, for example, that asking participants to donate money that they already earned may have yielded different results. Our policy support questions also differed from previous work by Iyengar [8, 9], which focused on support for welfare policy in the U.S. We utilized two scales to assess emotional responses to the stimuli—the first was the same scale that had been used in the research from which we adapted our stimulus material [22] and the second was a battery of emotional responses. While this approach mirrors what has been done in previous research, some studies have taken different approaches to assessing emotional responses, such as using self- and other-focused emotional scales [37] or assessing whether an individual believes that they will feel guilt or a warm glow [27]. It is possible that if we had used one of these alternative approaches to measuring emotional responses our results would have varied. In addition, we had all participants answer questions about emotional responses, individual behavior, and policy support. Though a central goal of this study was to reconcile the opposing findings concerning the effect of identified victims on different outcomes, it is possible that answers to the outcomes asked first (e.g. negative affect) may have affected responses to subsequent questions. Future research in this area may benefit by counter-balancing question order for the dependent variables or using separate experimental conditions to examine different dependent variables.

Some previous research [42] has suggested that individuals will only show a pattern of emotional responses aligned with the identified victim effect when the questions about emotional responses include information indicating that there will be a future request for a donation. In the present study, we followed a protocol similar to previous studies that found evidence for the identified victim effect [2,22], in which information about donating was included in the stimulus materials discussing the victim(s), but not in the *question prompt* for emotional responses. However, it is possible that our results may have differed if the actual question prompt for emotional responses noted that participants would later be asked about predispositions to donate.

While the present studies focused on general emotional responses, individual helping behavior, and policy support, they did not examine other concepts that may be related to the identified victim effect, such as internal mental imagery and perceived efficacy [23,43,44]. Research shows that generosity is influenced by the perceived efficacy of a particular helping behavior [44]; it is unclear if our outcomes–donations, policy support or volunteering–were perceived as being efficacious, which may have influenced our findings. It is also possible that appeals may interact with characteristics of the experimental respondents, as previous research has shown that respondents with different analytical skills [35] and motivations for charitable behavior [45] respond to appeals differently. Those with less analytical thinking may be more influenced by an appeal featuring a single identifiable victim and those seeking to make the greatest impact with their donation may respond more positively to statistical information. Thus, it may be that these individual characteristics are responsible for some of the conflicting findings in previous research and the null effects we see here.

While efforts were made to create realistic charitable appeals that were highly similar to previous work, it is possible that differences between the present appeals and the appeals used in previous research contributed to the non-significant results observed here. We also note that previous research has found that entitativity may moderate the effects of one versus many [3], such that if a group of identified individuals are presented as *related* to each other, they will elicit the same level of donations as when just a single individual is presented. While this information was not provided in the present studies and identifying information was presented in a way that was similar to previous research, it is possible that a cue such as the country of residence may have caused a “unitization” effect [46], thus attenuating the identified victim effect. Relatedly, previous research suggests that appeals for help are more successful when there is congruency between the entity in need and the beneficiary of help. For example, charity appeals featuring an identified victim promoted more helping behavior when the victim themselves, rather than the group they belonged to, was the beneficiary of help [47]. Because we did not vary the beneficiary in our studies, we are unable to test for a similar interactive effect.

Several studies have found that the identified victim effect is more robust when individuals are asked to help in-group victims, as compared to out-group victims [28,38]. It is possible that participants in the present study (residents of the United States) saw Kenyan individuals as part of a social outgroup, which may have influenced the results. However, we note that previous studies finding evidence for the identified victim effect have similarly used the paradigm of American participants considering the plight of African individuals in need [2,22].

It is also important to consider the power of the present studies. A priori, both Study 1 and Study 2 had a statistical power of .8 to detect an effect size of .11 or greater. However, a recent meta-analysis suggests that the overall effect size of the identified victim effect is .05 [48], meaning that despite the substantial number of participants per cell used in the present studies, it is possible that the studies were not sufficiently powered to detect very small effect sizes that may arise in this domain of research.

We recruited participants through online panels in order to achieve a large, diverse pool of participants for the studies. This also means that participants completed the experiment in a less controlled environment than if they had completed it in a lab, as has been done with some previous research in this area [2,22]. The less controlled environment may have led to distractions that can lower sympathy responses to help appeals [49]. Despite these limitations, the use of an online panel facilitated the presentation of realistic charitable appeals to a diverse sample with a greater number of participants per experimental cell than most previous work.

## Conclusion

The ultimate goal of many researchers in the area of prosocial psychology is to advance theoretical models that can be used to promote helping behavior. In many ways, the case of the individual victim effect offers an ideal illustration of the potential for decision research to have a substantive impact on the way journalists and philanthropic organizations communicate with the public. The findings of past research in this area are promising in their potential to help push individuals to feel compassion for and offer help to those in need. Yet in the present studies neither the identified victim [22] effect nor an effect in the opposite direction [8, 9] materialized, suggesting additional research is needed to better understand the nuances of victim presentations and examine how strategic communicators can meaningfully leverage such presentations to increase helping behavior. The fact that, in Study 1, the location of the individual in need did affect helping behavior suggests that in-group / out-group affiliations plays an important role in predispositions to help. Furthermore, future research may benefit by examining how additional factors such as perceived efficacy and attribution of responsibility may influence helping behavior when individual actions or policy interventions are being emphasized, and how this may be moderated by an in-group or out-group context. Finally, future research may benefit by continuing to identify boundary conditions of the phenomena detected in previous work and to determine the robustness of observed effects.

## Supporting information

S1 Fig Study 1 Stimulus–USA One. (JPG)

S2 Fig Study 1 Stimulus–USA Five. (JPG)

S3 Fig Study 1 Stimulus–USA Many. (JPG)

S4 Fig Study 1 Stimulus–USA One and Many. (JPG)

S5 Fig Study 1 Stimulus–Kenya One. (JPG)

S6 Fig Study 1 Stimulus–Kenya Five. (JPG)

S7 Fig Study 1 Stimulus–Kenya Many. (JPG)

S8 Fig Study 1 Stimulus–Kenya One and Many. (JPG)

S1 File Figure A. One Image. Figure B. Many Image. Figure C. No Image. Figure D. One Text. Figure E. Many Text.
(PDF)

S2 File Table A. Descriptive Statistics by Condition for Study 1. Table B. Zero-order correlations for Study 1 Dependent Variables. Table C. Descriptive Statistics by Condition for Study 2. Table D. Zero-order correlations for Study 2 Dependent Variables.
(PDF)

S3 File Data for Study 1. (SAV)

S4 File Data for Study 2. (SAV)
