# Supplementary figures and images for: The elusive power of the individual victim: Failure to find a difference in the effectiveness of charitable appeals focused on one compared to many victims
Source: PLoS One. 2018 Jul 18;13(7):e0199535. doi: 10.1371/journal.pone.0199535 (PMC6051573; doi:10.1371/journal.pone.0199535)

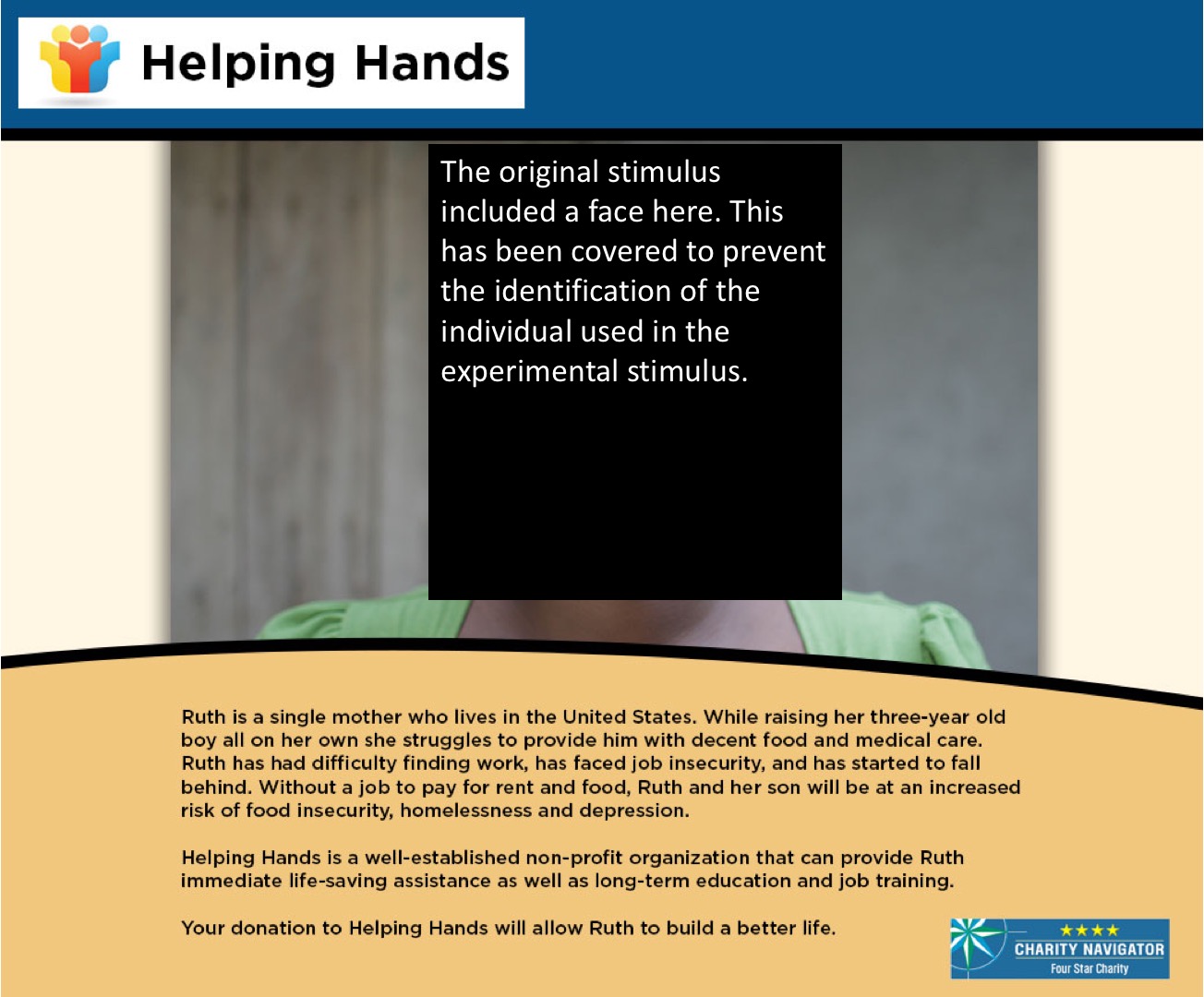

Supplement: S1 Fig — (JPG) [file pone.0199535.s001.jpg]

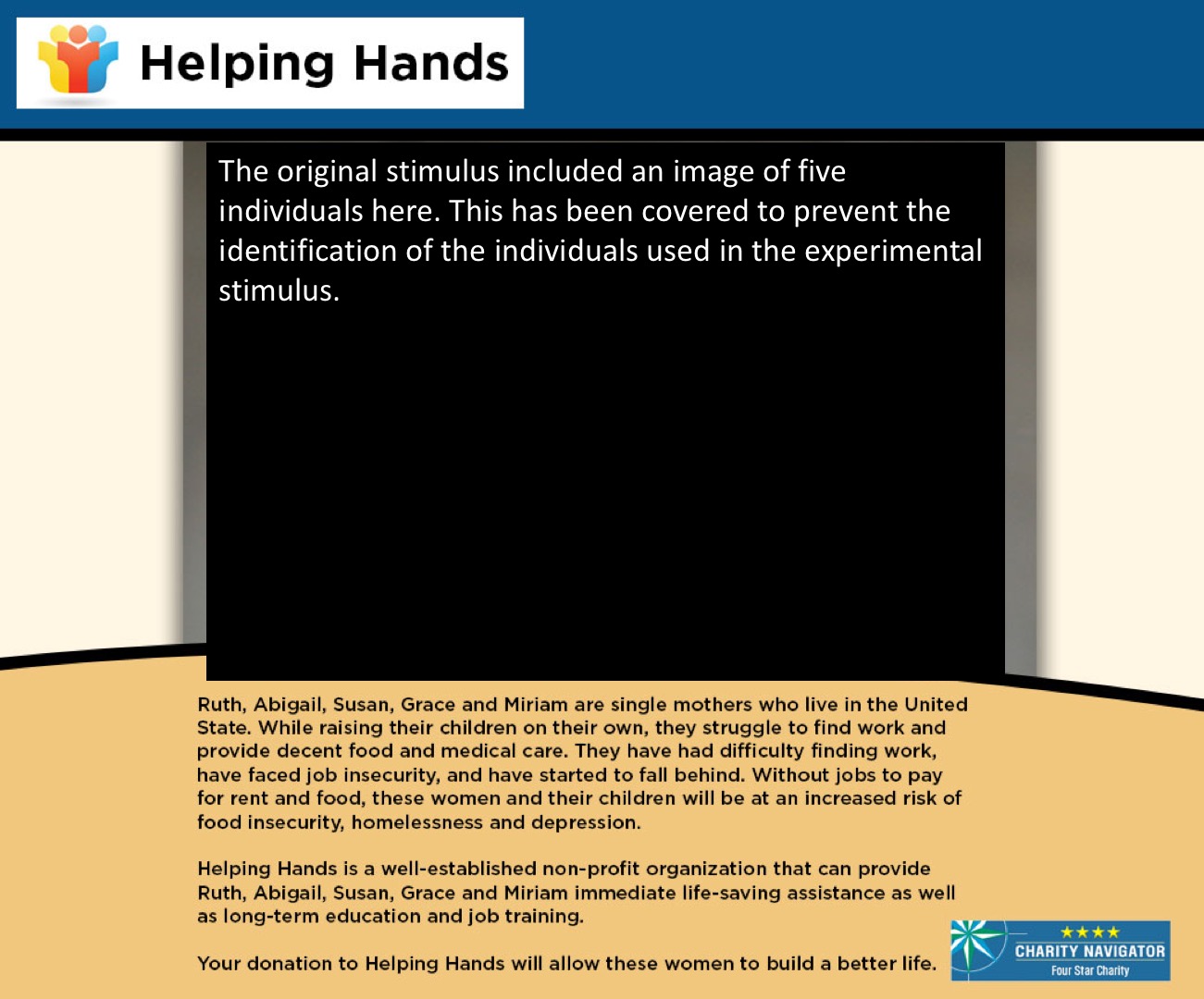

Supplement: S2 Fig — (JPG) [file pone.0199535.s002.jpg]

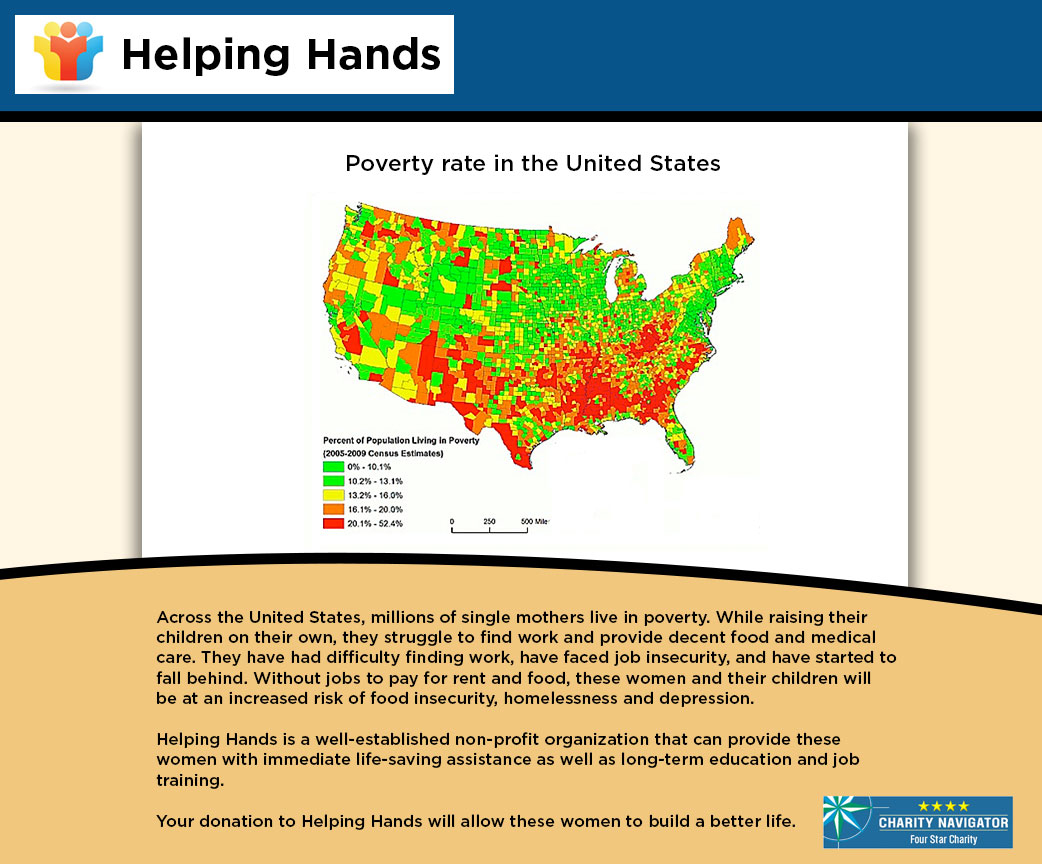

Supplement: S3 Fig — (JPG) [file pone.0199535.s003.jpg]

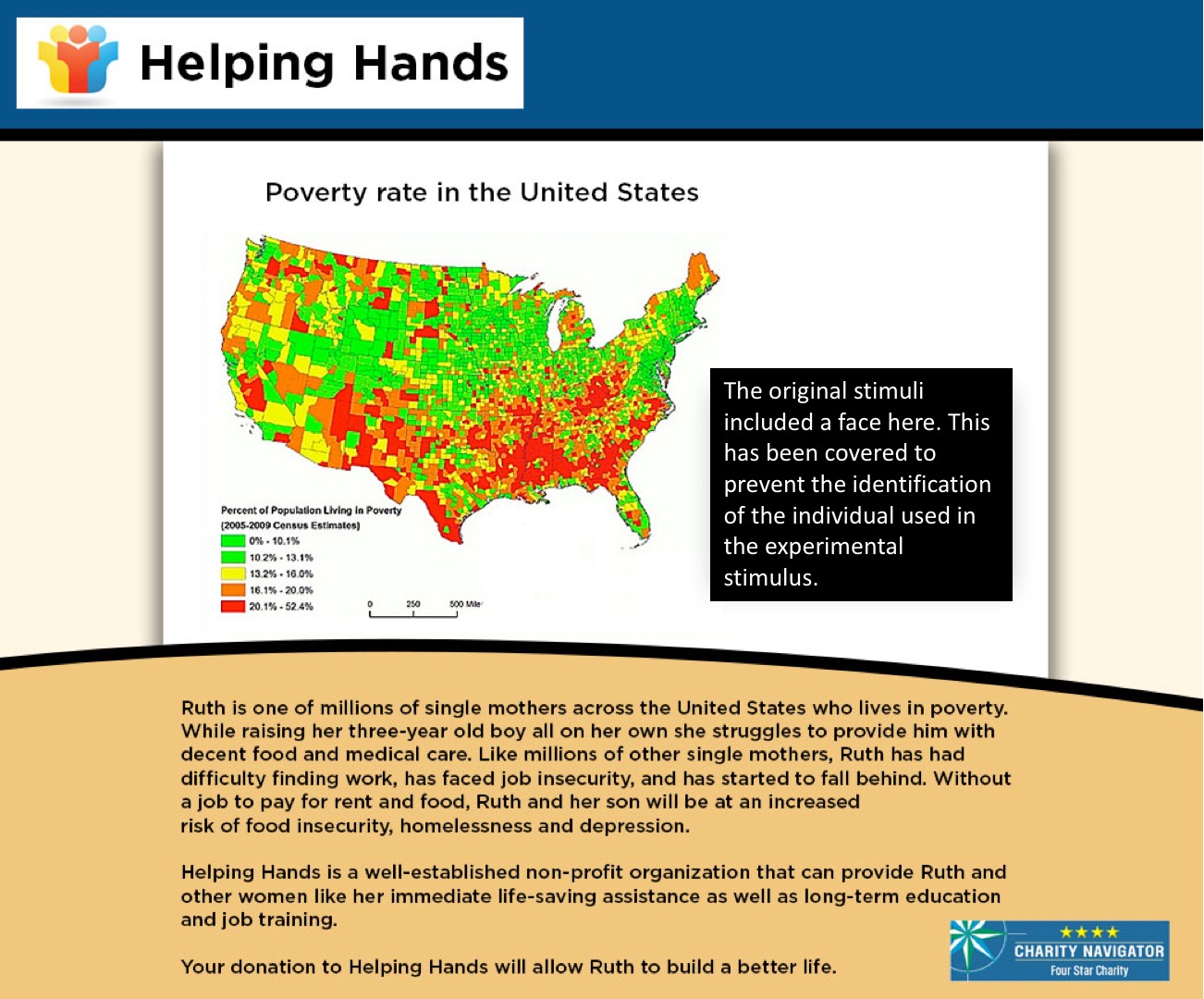

Supplement: S4 Fig — (JPG) [file pone.0199535.s004.jpg]

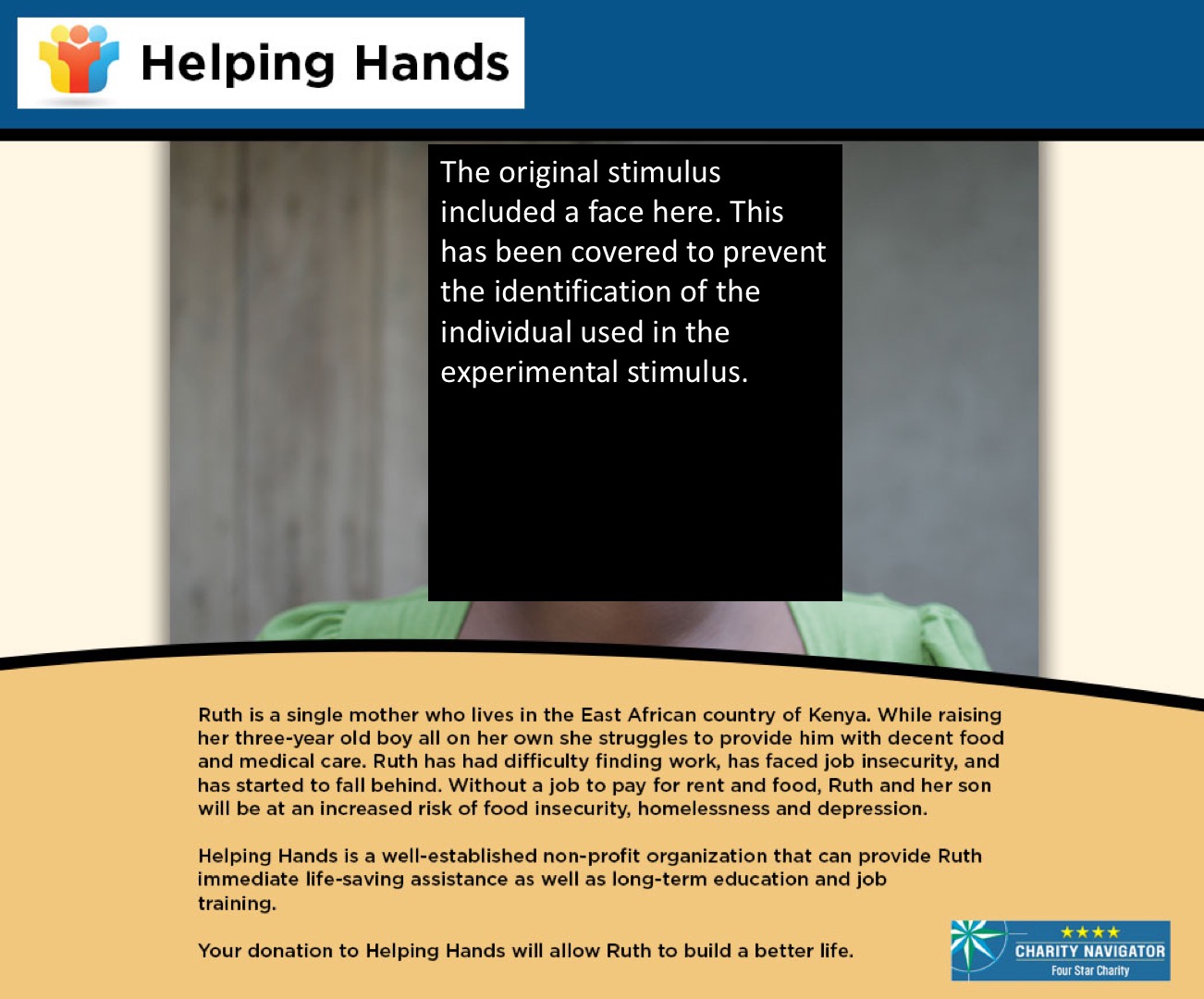

Supplement: S5 Fig — (JPG) [file pone.0199535.s005.jpg]

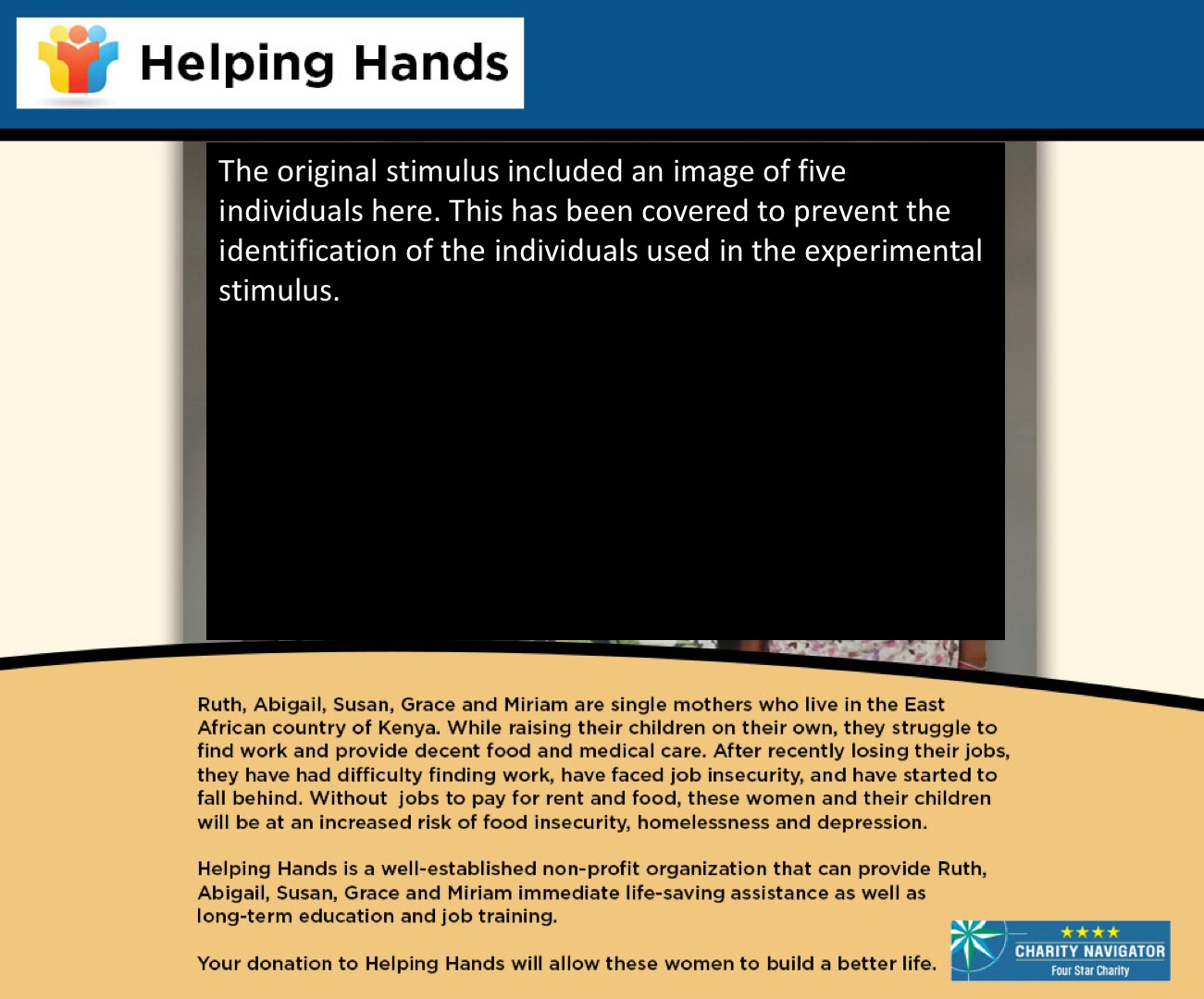

Supplement: S6 Fig — (JPG) [file pone.0199535.s006.jpg]

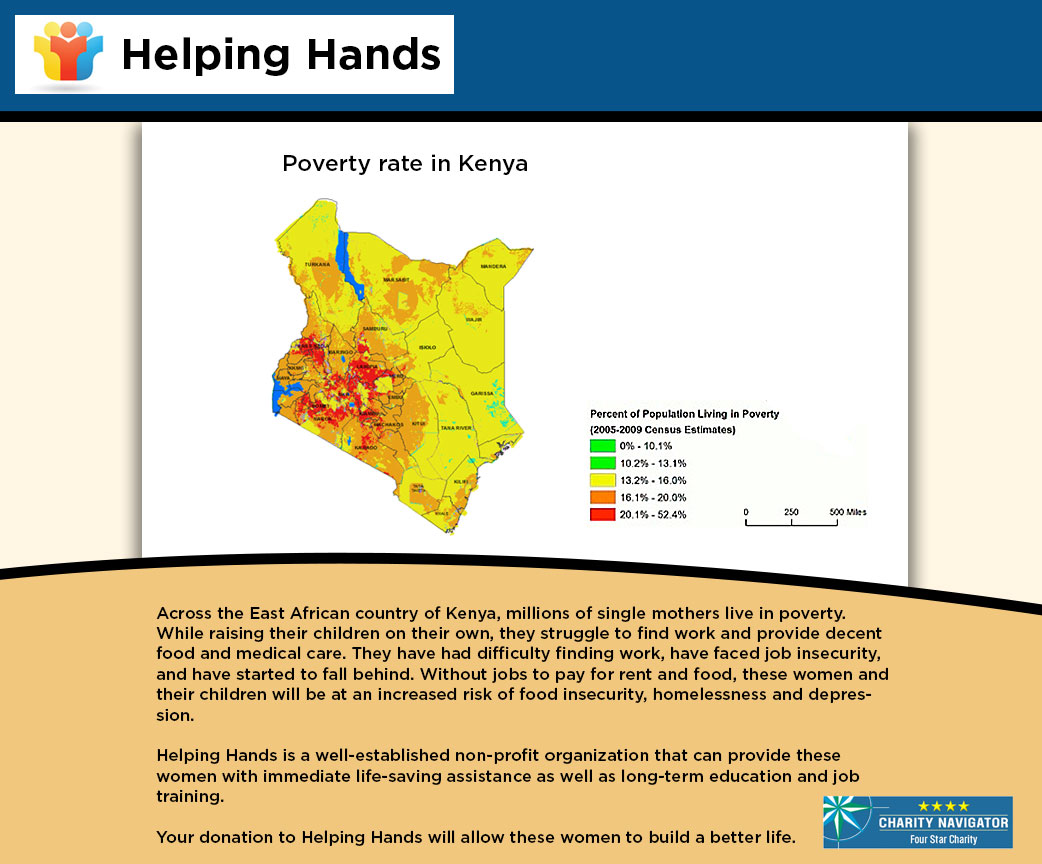

Supplement: S7 Fig — (JPG) [file pone.0199535.s007.jpg]

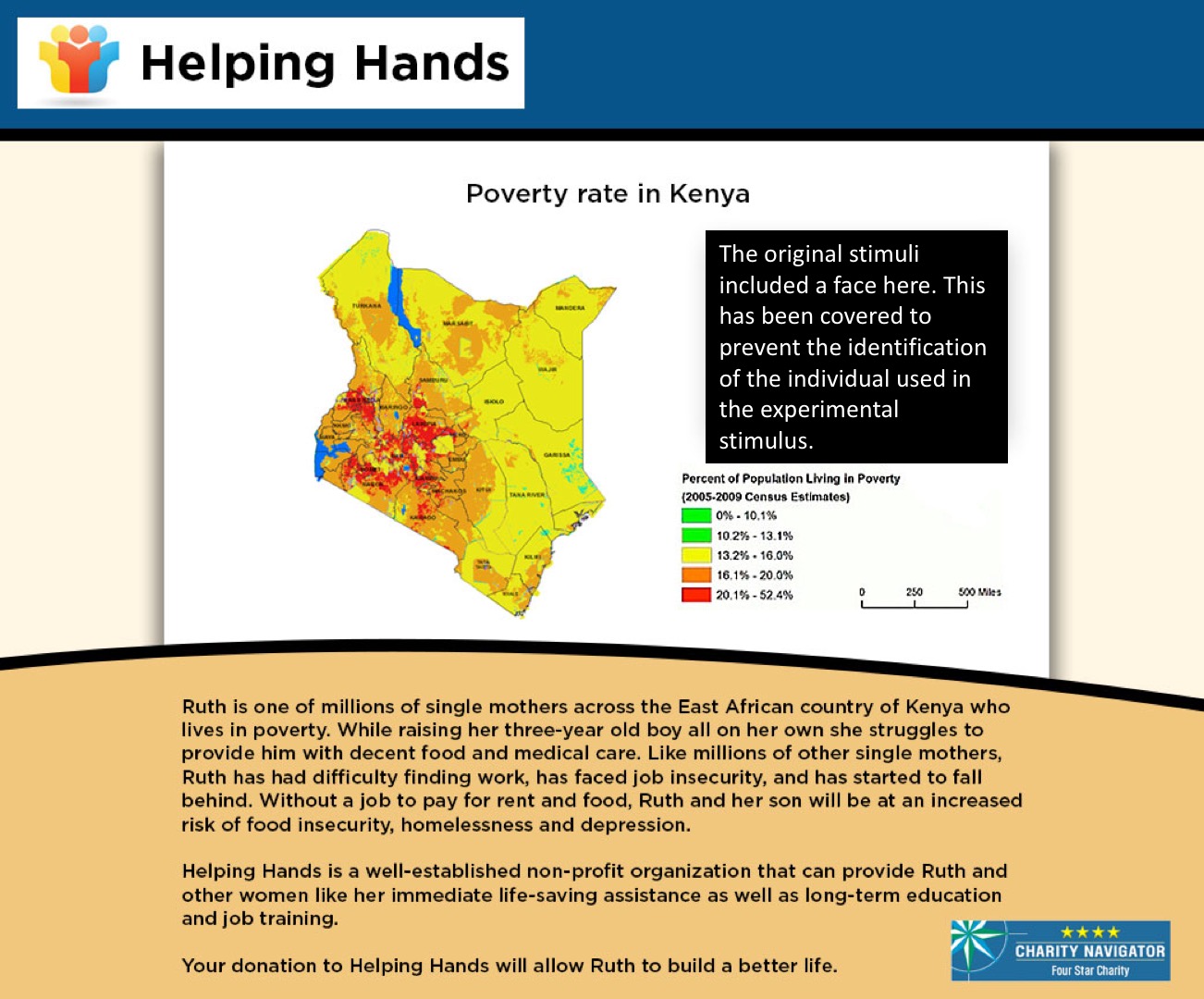

Supplement: S8 Fig — (JPG) [file pone.0199535.s008.jpg]
