# Supplementary material for: The elusive power of the individual victim: Failure to find a difference in the effectiveness of charitable appeals focused on one compared to many victims
Source: PLoS One. 2018 Jul 18;13(7):e0199535. doi: 10.1371/journal.pone.0199535 (PMC6051573; doi:10.1371/journal.pone.0199535)
Supplement: S1 File — Figure A. One Image. Figure B. Many Image. Figure C. No Image. Figure D. One Text. Figure E. Many Text. (PDF) [file pone.0199535.s009.pdf]

Supplementary Materials - S1 - Stimuli for Study 2

Image Factor (one, many or no image)

Figure A.

In the one image condition a single image of a girl in need was shown to participants. We are not publishing the photos use so the portrayed individuals cannot be identified.

Figure B.

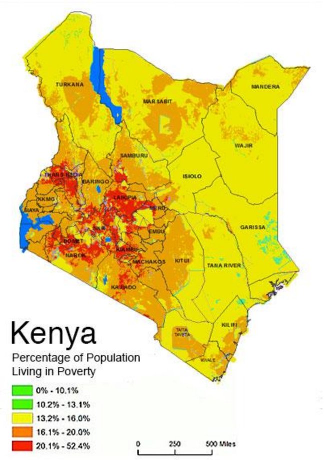

Figure C.

Text Factor (one or many):

Figure D.

Rokia, a 7-year-old girl from the east African country of Kenya, is desperately poor and faces a threat of severe hunger or even starvation. Her life will be changed for the better as a result of your financial gift. With your support, and the support of other caring sponsors, Save the Children will work with Rokia's family and other members of the community to help feed her, provide her with education, as well as basic medical care and hygiene education.

Figure E.

Girls across the east African country of Kenya are desperately poor and face the threat of severe hunger or even starvation. Their lives will be changed for the better as a result of your financial gift. With your support, and the support of other caring sponsors, Save the Children will work with these girls' families and other members of their communities to help feed them, provide them with education, as well as basic medical care and hygiene education.
