# Supplementary material for: The elusive power of the individual victim: Failure to find a difference in the effectiveness of charitable appeals focused on one compared to many victims
Source: PLoS One. 2018 Jul 18;13(7):e0199535. doi: 10.1371/journal.pone.0199535 (PMC6051573; doi:10.1371/journal.pone.0199535)
Supplement: S2 File — Table A. Descriptive Statistics by Condition for Study 1. Table B. Zero-order correlations for Study 1 Dependent Variables. Table C. Descriptive Statistics by Condition for Study 2. Table D. Zero-order correlations for Study 2 Dependent Variables. (PDF) [file pone.0199535.s010.pdf]

Supplementary Materials – S2 – Tables for Descriptive Statistics and Correlations for Studies 1 and 2

Table A.

	One victim		Five victim		Many victims		One & Many victims	
**Location = United States**								
	Mean	SD	Mean	SD	Mean	SD	Mean	SD
Feelings	3.40	0.97	3.20	1.04	3.49	0.97	3.47	0.94
Negative affect	4.08	0.98	3.84	1.06	3.99	0.95	4.08	0.97
Donation Willingness	0.75	0.44	0.58	0.50	0.77	0.42	0.70	0.46
Donation Amount	14.74	15.43	11.21	14.39	13.67	13.79	13.36	13.12
Policy Support	4.52	1.05	4.54	1.03	4.72	1.02	4.74	1.01
Willingness to volunteer	4.05	1.15	3.84	1.04	4.02	1.15	3.97	1.16
**Location = Kenya**								
	**One victim**		**Five victim**		**Many victims**		**One & Many victims**	
	Mean	SD	Mean	SD	Mean	SD	Mean	SD
Feelings	3.24	0.94	3.15	0.98	3.21	0.93	3.13	1.00
Negative affect	3.92	0.89	3.98	1.05	3.85	1.10	3.89	1.00
Donation Willingness	0.71	0.45	0.66	0.47	0.69	0.47	0.61	0.49
Donation Amount	12.88	14.52	12.82	13.15	13.14	13.43	10.94	13.63
Policy Support	3.77	1.01	3.66	1.11	3.69	1.21	3.70	1.13
Willingness to volunteer	3.64	1.08	3.68	1.04	3.68	1.19	3.65	1.30

Table B.

	1.	2.	3.	4.	5.	6.
1. Feelings	-					
2. Negative affect	.715**	-				
3. Donation Willingness	.479**	.345**	-			
4. Donation Amount	.442**	.302**	.628**	-		
5. Policy Support	.563**	.426**	.328**	.286**	-	
6. Willingness to volunteer	.698**	.513**	.493**	.416**	.534**	-

Note. Coefficients are zero-order Pearson correlations. $N = 924$. $^{**}p < .01$.

Table C.

	One Victim					
	**Individual Child Image**		**Map Image**		**No Image**	
	Mean	SD	Mean	SD	Mean	SD
Feelings	3.20	0.99	3.26	0.96	3.14	1.12
Negative affect	4.12	1.15	4.18	1.00	4.03	1.22
Donation Willingness	0.41	0.49	0.41	0.49	0.43	0.50
Donation Amount	6.59	11.73	7.85	13.51	9.14	14.87
Policy Support	3.96	1.50	3.95	1.53	3.89	1.60
Willingness to volunteer	3.36	1.45	3.55	1.39	3.40	1.52
	**Many Victims**					
	**Individual Child Image**		**Map Image**		**No Image**	
	Mean	SD	Mean	SD	Mean	SD
Feelings	3.27	0.94	3.05	1.09	3.23	1.08
Negative affect	4.13	1.03	3.92	1.13	4.04	1.13
Donation Willingness	0.43	0.50	0.41	0.49	0.47	0.50
Donation Amount	8.19	14.13	7.37	14.11	8.15	13.72
Policy Support	3.92	1.46	3.63	1.60	3.89	1.59
Willingness to volunteer	3.50	1.36	3.23	1.39	3.36	1.42

Table D.

	1.	2.	3.	4.	5.	6.
1. Feelings	-					
2. Negative affect	.730**					
3. Donation Willingness	.617**	.418**				
4. Donation Amount	.496**	.320**	.666**			
5. Policy Support	.628**	.519**	.473**	.408**		
6. Willingness to volunteer	.710**	.556**	.604**	.525**	.681**	-

Note. Coefficients are zero-order Pearson correlations. $N = 1085$. $^{**}p < .01$.
